# Regulatory T Cells in Allogeneic Stem Cell Transplantation

**DOI:** 10.1155/2013/608951

**Published:** 2013-05-08

**Authors:** Maria Michael, Avichai Shimoni, Arnon Nagler

**Affiliations:** ^1^Hematology Division & Bone Marrow Transplantation, Chaim Sheba Medical Center, Sackler Medical School, Tel Aviv University, Tel Aviv 52621, Israel; ^2^Hematology Division & Cord Blood Bank, Chaim Sheba Medical Center, Tel Hashomer, Tel Aviv 52621, Israel

## Abstract

Growing evidence suggests that cellular adoptive immunotherapy is becoming an attractive though challenging approach in regulating tumor immunity and alloresponses in clinical transplantation. Naturally arising CD4+CD25+Foxp3+ regulatory T cells (Treg) have emerged as a key component in this regard. Over the last decade, a large body of evidence from preclinical models has demonstrated their crucial role in auto- and tumor immunity and has opened the door to their “first-in-man” clinical application. Initial studies in clinical allogeneic stem cell transplantation are very encouraging and may pave the way for other applications. Further improvements in Treg *ex vivo* or *in vivo* expansion technologies will simplify their global clinical application. In this review, we discuss the current knowledge of Treg biology and their potential for cell-based immunotherapy in allogeneic stem cell transplantation.

## 1. Introduction

In recent years, the great progresses in our understanding of the basic processes that control immune tolerance, as well as the more recent characterization of naturally arising CD4+CD25+Foxp3+ regulatory T cells (Treg), that tip the balance between auto- and tumor immunity, opened the door to their therapeutic application, either by enhancing their activity in autoimmune diseases [1–3], allograft rejection [3], and graft-versus-host disease (GVHD) [4, 5] or by blocking their suppressive activity in tumor immunity [6] and in vaccine development [7]. Treg therapy has the promise of avoiding many of the toxicities observed with current drug regimens. However, many issues on the homeostasis and function of human Treg still need to be addressed. The development of new markers and technologies for Treg identification, antigen-specific isolation, and *in vitro* or *in vivo* expansion by specific stimulation will help to “unlock the power” of Treg and devise novel therapeutic strategies to control untoward immune responses.

In this review, we discuss the current knowledge of Treg biology and their potential for cell-based immunotherapy in allogeneic stem cell transplantation.

## 2. Biology of Treg

Human natural Treg (nTreg) derive from thymus and are characterized by the coexpression of CD4, high levels of surface CD25 (also known as interleukin-2 receptor *α* (IL-2R*α*)), and intracellular expression of a master switch transcription factor called forkhead box P3 (Foxp3) [8]. Treg can be distinguished from activated CD25+ conventional T cells (Tcon) by their low or absent surface expression of CD127 (also known as IL-7R) [9, 10].

Induced or adaptive Treg (iTreg) can be generated from naïve T cells *in vitro* via specific stimulation conditions or induced in the peripheral lymphoid organs *in vivo*, with transforming growth factor *β* (TGF*β*) playing a pivotal role [11–13].

As demonstrated a decade ago, the transcription factor Foxp3 is indispensable for both nTreg and iTreg development and suppressive function [8, 14, 15]. Absence of functional Foxp3 protein due to mutations in the *Foxp3 gene * results in the development of severe autoimmune disorders as can be observed in the “scurfy” mouse mutant [14] and patients suffering from immune dysregulation, polyendocrinopathy, enteropathy, and X-linked syndrome (IPEX) [15]. Very recent data revealed another particularly important intracellular protein for proper Treg development, the Helios transcription factor, a member of the Ikaros family, that has been shown to upregulate expression of Foxp3 protein. Furthermore, constant Helios expression throughout Treg expansion can keep Foxp3 highly expressed, which results in a more stable population [16, 17].

Recent studies suggest that nTreg are more stable compared with iTreg. This is related to their different DNA methylation profiles and to other epigenetic regulations of *Foxp3* [18–21]. In particular, a conserved region upstream of exon 1 within the *Foxp3* locus, the so-called *Treg-specific demethylation region (TSDR)*, is completely demethylated in nTreg but fully methylated in Tcon and iTreg [21–23]. The TSDR is a transcription factor binding site, and its enhancer function stabilizes Foxp3 expression in Treg [22, 24].

 Thus, due to unstable Foxp3 expression, *in vitro* differentiated human iTreg might not be stable phenotypically and functionally, implying that *in vivo* transfer of iTreg for therapeutic purposes may give unexpected results and should be considered with caution [25].

Significant progress has been made over the last few years in delineating the mechanism of suppression exerted by nTreg [26]. Numerous putative mechanisms have been proposed in the literature that can be subdivided into two categories: dependent on cell-cell contact and/or mediated by cytokines. *In vitro*, nTreg were shown to inhibit the activation of effector CD4+CD25− T cells predominantly by cell-cell contact dependent mechanisms. nTreg express on their surface important molecules for their suppressive function such as cytotoxic-T-lymphocyte-associated-antigen-4 (CTLA-4), membrane-bound TGF*β* latency-associated peptide (LAP), glucocorticoid induced tumour necrosis factor receptor (GITR), CD4-related lymphocyte-activation-gene-3 (LAG-3), galectin-1, and CD39. Moreover, after activation, human nTreg were shown to be able to directly kill CD4+ and CD8+ T cells via the secretion of perforin and granzyme B. The role of regulatory cytokines such as IL-10, TGF*β*, and, more recently, IL-35 in nTreg-mediated suppression of immune pathologies has mainly been described in *in vivo* experimental models [25].

Also, IL-2 is crucial for nTreg homeostasis, as these cells are highly dependent on exogenous IL-2 for growth *in vitro* and *in vivo *and for their peripheral maintenance and competitive fitness [27, 28]. Moreover, the high expression of CD25 empowers Treg to “consume” local IL-2 and therefore starve actively dividing effector T cells by depleting the IL-2 they need to survive [26].

In addition to directly affecting effector T cell function, nTreg can modulate the maturation and/or function of dendritic cells required for effector T cell activation [26].

To summarize, Treg are a specialized subpopulation of T cells that suppress the activation, expansion, and function of other T cells, thereby maintaining homeostasis through a fine balance between reactivity to foreign and self-antigens. More importantly, strategies to expand this population by *ex vivo* culture *or in vivo* by specific stimulation will help to devise novel therapeutic strategies to control untoward immune responses.

In the allogeneic stem cell transplantation setting, preclinical models demonstrated (as discussed below) that graft-versus-host disease (GVHD) prevention and transplantation tolerance require tipping the balance in favour of Treg against effector T cells.

## 3. Treg Suppress GVHD in Murine Models

A number of groups have demonstrated that in aggressive murine GVHD models, where bone marrow and GVHD-inducing Tcon were transplanted across complete major histocompatibility complex (MHC) class I and II barriers, lethal acute GVHD was prevented by donor Treg, if cotransplanted at 1 : 1 ratio with Tcon [4, 5, 29]. This 1 : 1 ratio was also then evaluated for the impact of Treg on Tcon-associated beneficial graft-versus-tumor (GVT) effect, while maintaining protection from GVHD [30]. Cotransfer of donor Treg induced a profound suppression of the proliferation of Tcon in secondary lymphoid organs by >90% at day 7 and GVHD target tissues at this ratio. *In vitro* analysis of splenic Tcon on day 5 from different experimental groups for their phenotype, activation markers, and cytokine production has shown that Treg do not inhibit activation and functional maturation of Tcon.

In two independent tumor models, including the leukemia line A20, which infiltrates the bone marrow, and BCL1 lymphoma, which primarily invades liver and spleen, Treg did not interfere with the GVT activity of Tcon but permitted the elimination of tumors from all these compartments, resulting in long-term survival of the hosts.

The presence of Treg therefore does not abrogate the activation of Tcon or their cytolytic response. The investigators hypothesized that the significant decrease in Tcon proliferation by Treg controlled acute GVHD, which generally requires a strong alloreactive response. However, this decrease is not complete, and the remaining Tcon are sufficient to effectively mount a GVT effect [30]. 

Additional studies [31] demonstrated that regulatory suppression of GVHD by murine Treg preserved thymic and lymphoid architecture of the host and can thereby accelerate posttransplant T cell immune reconstitution. 

## 4. Treg and Induction of Transplantation Tolerance

Treg have been shown to mediate transplantation tolerance in murine models of skin, solid organ transplantation [32–34], and, more recently, antigen-specific tolerance to bone marrow allografts. In a semiallogeneic murine model [35], transplanted bone marrow was protected from rejection by host T cells, through injection of host Treg preactivated *in vitro* against donor antigen-presenting cells (APCs). When a third-party bone marrow was cotransplanted, host preactivated Treg preferentially augmented donor chimerism, as assessed 2 weeks after transplantation (most notably at lower Treg to effector T cell ratios). 

In another murine MHC-mismatched bone marrow transplantation (BMT) model [36], cotransplantation of donor Treg into sublethally irradiated recipients resulted in decreased rejection of both lineage-committed and multipotential donor hematopoietic progenitors within the first week of the transplantation. Enhanced engraftment with Treg was further shown with increased long-term donor chimerism in animals that received Treg compared with those that received bone marrow transplant only. Importantly, recipients of Treg in this model demonstrated tolerance to host and donor antigens but mounted responses to third-party antigens such as skin allografts and *in vitro* polyclonal T and B cell stimulations. 

Unlike generalized immunosuppressive regimens, Treg are long-lived and functional in a dominant and antigen-specific manner. Thus, therapeutic infusion of Treg has the potential to induce long-term donor-specific tolerance without impeding desired immune responses to pathogens and tumors in transplant patients. 

## 5. Treg and Tumor Progression

There is accumulating evidence that Treg may also modulate host T cell activity against tumor-associated antigens, thereby facilitating tumor escape from immunological control. Treg were shown to be expanded in murine tumor models [37]. Moreover, their deletion reinstated an efficient antitumor immune response leading to complete tumor regression [38–40]. 

Several reports have demonstrated that Treg are expanded in patients with solid tumors [41–45], B cell chronic lymphocytic leukemia (CLL) [46–48], monoclonal gammopathy of undetermined significance (MGUS), and multiple myeloma (MM) [49, 50], Hodgkin lymphoma [51, 52], or non-Hodgkin lymphomas (NHLs) [53], but little is known about the differentiation, origin, antigen specificity, and mechanisms of expansion of Treg in cancer patients. 

In CLL, a direct correlation between higher Treg numbers and more aggressive clinical-biological features of the disease, as well as with disease progression, has been described [54]. Moreover, the percentage of circulating Treg appears able to predict the time to first treatment in low-risk patients, thus emerging as a useful biomarker with prognostic power [55, 56]. Interestingly, when patients with CLL were treated with fludarabine frequencies of Treg were normalized, despite an initial transient increase [57]. In another study [58], treatment with lenalidomide resulted, after a transient increase in the percentage of Treg after 3 cycles, in a significant decrease in Treg after 15 cycles of therapy. These data are consistent with the *in vitro* inhibitory effect of lenalidomide on Treg [59] and suggest a mechanism by which this drug may help overcome an important barrier to tumor-specific immunity in cancer patients.

In a recent study in newly diagnosed MM patients [50], increased frequencies of suppressive Treg correlated with lower overall survival of the patients, suggesting a certain role of Treg in facilitation of disease progression and infectious complications. Specifically, patients with higher percentages of Treg (equal or above median 6.16%) lived shorter when compared with those with lower frequencies of Treg (median overall survival 21 months versus not-reached, *P* = 0.013, at median follow-up of 32 months). Interestingly, Treg frequencies were not influenced after chemotherapy with novel antimyeloma agents (CTD regimen: cyclophosphamide, thalidomide, and dexamethasone or MPT regimen: melphalan, prednisone, and thalidomide) nor autologous stem cell transplantation reduced Treg significantly.

Alternatively, suppressive Treg might actually play a beneficial role in Hodgkin lymphoma (HL), which is characterized by a chronic background inflammatory response, important for the proliferation and survival of Hodgkin-Reed-Sternberg (HRS) cells. The frequency of Foxp3+ T cells was determined in lymphoma-afflicted lymph nodes (LNs). Low frequencies of Foxp3+ cells and high frequencies of cytotoxic T/NK lymphocytes (CTLs) in the reactive background of LNs correlated with poor overall survival [52, 60]. However, costaining for Foxp3 and CD4 or CD25 was not performed, limiting the significance of this finding because Foxp3 expression in humans might not be confined to Treg only [61]. 

As already outlined, a correlation of increased Treg with greater disease burden and poorer overall survival has been reported. Treg are able to recognize tumor-associated self-antigens and to control natural T cell responses against various cancer antigens, which may explain the failure of many cancer vaccines [62]. In addition, a therapeutic cancer vaccine could induce tumor-specific Treg that blunt the expansion and function of antitumor T cells [62]. In line with these results, in an attempt to improve vaccination efficacy against foreign antigens and to break tolerance against self-tumor antigens, various approaches have been developed to deplete or inhibit the activity of Treg [63, 64].

 A recent phase I study [65] demonstrated that vaccination with a myeloma-specific vaccine, generated by the fusion of patient-derived MM cells with autologous dendritic cells (DCs), resulted in the expansion of tumor reactive lymphocytes and disease stabilization in a majority of patients with advanced myeloma. Of note, the majority of patients in this trial exhibited a dampening of immunologic response 6 months after vaccination, which suggests the downmodulation of antitumor immunity. Immunosuppressive features prevalent in MM patients, including the increased presence of Treg, likely interfere with vaccine efficacy. In this regard, the same group examined the effect of lenalidomide *in vitro* on the response to the MM tumor vaccine [7]. Stimulation with DC/MM fusions in the presence of lenalidomide resulted in enhanced expansion of T cell expressing IFN-*γ*, decreased levels of Treg, and, most significantly, an increased capacity of vaccine stimulated T cells to lyse autologous MM cells. Hence, lenalidomide may create an ideal platform for myeloma-specific immunotherapy that acts synergistically with therapeutic vaccination with DC/MM fusions to induce myeloma-specific immunity.

In conclusion, therapeutic vaccination in conjunction with Treg depletion as a mean to augment vaccine response remains an area of further investigation.

## 6. Treg for Prevention and Treatment of GVHD: First-In-Man Clinical Trials

Given the striking results in murine GVHD and bone marrow graft rejection models, the ready availability of donor Treg together with the known and transient risk period for adverse consequences from alloreactive T cells and the high degree of morbidity and mortality associated with allogeneic stem cell transplantation (SCT), it is not surprising that GVHD prevention has emerged as the first clinical application for human Treg.

 In the “first-in-man” clinical trial [66], *in vitro* expanded Treg (average 64% Foxp3+ after expansion) derived from partially HLA-matched third-party umbilical cord blood units were used in 23 patients undergoing double-cord blood transplantation. Treg were administered on day +1 post-transplant (fresh) (doses ranged from 1 × 10^5^ to 3 × 10^6^/kg) and additionally on day +15 (3 × 10^6^/kg) in 13 patients, using cryopreserved Treg expanded from the same cord blood unit. The rates of GVHD and infectious complications were compared with those from 108 historical controls. Importantly, no increase in opportunistic infections and no Treg-related acute toxicities were observed. The authors did report a reduced incidence of grades II–IV acute GVHD (43% versus 61%, *P* = 0.05) in the trial group, but efficacy for the prevention of GVHD could not be demonstrated definitely in this phase I safety and feasibility trial, as standard pharmacologic prophylaxis was coadministered (cyclosporine A/mycophenolate mofetil or sirolimus/mycophenolate mofetil).

In another small phase I safety and feasibility trial [67], Edinger and Hoffmann transfused freshly isolated donor Treg into 9 patients with high risk of leukemia relapse after allogeneic SCT. In this preemptive donor lymphocyte infusion strategy, up to 5 × 10^6^ cells/kg (>50% Foxp3+) were administered after the cessation of pharmacologic GVHD prophylaxis (within a year after SCT). After an observation period of 8 weeks, additional Tcon were administered at the same dose to promote GVL activity. No Treg-related acute toxicity was observed; neither GVHD nor opportunistic infections or early disease relapses occurred after Treg transfusion, despite the absence of pharmacologic immunosuppression. By design, this trial was not suited to prove the efficacy of Treg for the prevention of GVHD because of the low patient number and the lack of control group.

A more recent study [68] demonstrated for the first time that adoptive immunotherapy with freshly purified Treg counteracts the GVHD potential of a high number of donor Tcon in patients receiving an HLA-haploidentical graft. In this trial, 28 patients undergoing haploidentical SCT received freshly isolated donor Treg (average 69% Foxp3+; *n* = 24 with 2 × 10^6^/kg, *n* = 4 with 4 × 10^6^/kg) on day −4, which was followed by transfer of a highly purified CD34+ stem cell graft together with Tcon. Patients did not receive any prophylactic immunosuppression. For safety reasons of this pilot trial, the first group of 4 patients received only 25% Tcon compared to Treg. Because none of these first 4 patients developed acute GVHD, Tcon were then escalated to 50% of the Treg dose. Rapid and stable engraftment was seen, and, surprisingly, only 2 of 26 evaluable patients developed grades II–IV acute GVHD, while the majority of patients remained free of clinically relevant GVHD. At a median follow-up of 11.2 months, no patient developed chronic GVHD. When compared to a dataset of 152 patients receiving haploidentical SCT without Treg transfer, this approach promoted lymphoid reconstitution and improved immunity to opportunistic pathogens. These first clinical data suggest that donor Treg infusion prevents acute GVHD after allogeneic SCT in humans, because lethal GVHD would have otherwise been expected in all patients after the administration of such high donor Tcon numbers in the absence of pharmacologic immunosuppression. Thus, these results are highly encouraging and now demand confirmation in randomized controlled multicenter trials.

Clinical trials exploring the efficacy of Treg for the treatment of GVHD are much more challenging than prevention trials, and therefore it is not surprising that evidence is still sparse. 

High Treg numbers and maximum Treg purity would be required to avoid aggravation of GVHD by contaminating Tcon. Since Treg have to be isolated from the stem cell donor and require two to three weeks *in vitro* expansion, cell production may often be too slow for patients with severe and rapidly progressive disease. 

In a recent anecdotal report [69] it was suggested that the transfer of *in vitro* expanded donor Treg (90% Foxp3+; single dose of 1 × 10^5^/kg) contributed to the amelioration of chronic GVHD in a single patient and allowed mycophenolate mofetil (MMF) withdrawal and a reduction in steroids. A second patient treated with higher Treg numbers (total dose of 3 × 10^6^/kg over three infusions) for treatment-resistant acute GVHD had no benefit. The last Treg infusion for that patient contained only 40% Foxp3+ cells, and it is questionable whether such a cell product should have been administered in a life-threatening disease caused by donor Tcon.

Edinger and Hoffmann [67] have also transfused *in vitro* expanded Treg (>95% Foxp3+) in a small number of patients with treatment-resistant acute GVHD and found that these cells survive *in vivo* and may ameliorate severe acute gastrointestinal GVHD. Clinical trials are clearly warranted to test the therapeutic potential of donor Treg infusion for ongoing GVHD.

## 7. Treg and Infectious Complications

Like all therapies, clinical use of *ex vivo* expanded Treg is associated with potential risks. One issue to be investigated in future studies is whether adoptive immunotherapy with Treg compromises general immunity, blunting responses to infectious agents.

In animal models, it has been demonstrated that Treg not only prevent GVHD but also enhance immune reconstitution after bone marrow plus Tcon transplant, by preventing GVHD-induced damage of the thymus and secondary lymphoid organs, thus allowing protection against lethal cytomegalovirus (CMV) infection [31].

Limited safety data have been obtained from initial clinical trials. As described above, the phase I clinical trial by Brunstein et al. [66] reported that Treg infusion in patients who had undergone double umbilical cord blood transplantation did not increase the incidence of fungal, bacterial, or viral infections compared with the control group. 

In the clinical trial by di Ianni et al. [68], Treg infusion prior to haploidentical transplantation did not inhibit immune reconstitution. CD4+ and CD8+ cell counts achieved sustained levels quickly, and high frequencies of pathogen-specific CD4+ and CD8+ T cell precursors were detected as early as 2 months after transplantation. Strikingly, the prevention of CMV disease was markedly improved, with no CMV-related deaths, an improvement over 40% of nonleukemic deaths caused by CMV that had previously been reported by this group. Furthermore, seven patients were vaccinated against influenza 3–14 months post-transplant, and five acquired protective antibody titers. It was hypothesized that, as in animal models, in a postconditioning inflammatory environment, Treg are activated by recipient APCs, block alloreactive T cells in an antigen-specific fashion, and allow the expansion of nonalloreactive T cells, which ensures long-term immunity.

## 8. Drugs That Potentiate Treg

A significant challenge for the use of nTreg in the clinic is the difficulty in isolating sufficient numbers of Treg for clinical application, since circulating numbers of Treg in the peripheral blood are limited (5%–10% of CD4+ T cells) [70, 71]. Current research focuses on the development of large-scale expansion protocols for Treg with higher cell yields [72–74].

The use of granulocyte-colony-stimulating-factor (G-CSF) may also be considered to increase Treg mobilization into the peripheral blood for potential extraction during leukapheresis [75]. Under steady state, human bone marrow contains a large fraction of CD4+CD25+Foxp3+ T cells with regulatory function [76]. Recent data suggests that donor Treg after G-CSF stimulated stem cell mobilization retain their potent suppressive and stable phenotype, and, thus, the adoptive transfer of donor Treg after G-CSF stimulation appears to be feasible and safe [75]. The isolation of donor Treg from the stem cell graft will simplify their global clinical application.

It has been recently shown that expansion of adoptively transferred Treg *in vivo* is critical for their GVHD suppressive activity [77]. This would alleviate the need for cumbersome *ex-vivo* manipulations, thus rendering the therapy more clinically applicable. 

Recent data suggests that T cell depletion protocols used to induce tolerance in clinical transplantation, for example, by using total lymphoid irradiation (TLI) and antithymocyte globulin (discussed below) as conditioning regimen [78, 79], alter the balance of residual host T cells subsets to enrich host innate regulatory natural killer T cells (NKT cells) that cooperate with donor Treg and induce IL-4 dependent *in vivo* Treg expansion.

Rapamycin, a small molecule inhibitor of the Akt/mTOR pathway used for the prophylaxis and treatment of GVHD, has been shown *in vitro* to selectively expand or preferentially preserve Treg over Tcon [80], thus attenuating GVHD also by shifting the balance of aggressive to protective type alloimmunity. Data from murine *in vivo* SCT models support the observation of Treg-supportive effects of rapamycin [81], including increased generation of thymic Treg [82] and infiltration into GVHD target organs [83]. 

As stressed before, *in vivo* homeostasis and expansion of Treg are highly dependent on IL-2. Thus, IL-2 is also investigated as a putative agent for selective expansion of Treg [84]. Recently, a phase I escalation study of IL-2 administration to 29 patients with active steroid-refractory chronic GVHD was performed [85]. Administration of daily subcutaneous low-dose IL-2 rapidly induced preferential and sustained Treg expansion, reversed advanced fibrotic and sclerotic manifestations of chronic GVHD in a substantial proportion of patients, and permitted a substantial reduction in glucocorticoid dose. 

Also, the combination of rapamycin plus IL-2 appears to be more effective than rapamycin alone in the prevention or suppression of GVHD by *in vivo* expansion of nTreg, enhanced conversion of iTreg by IL-2, and inhibition of effector T cells [86].

It has also been shown that rabbit-derived anti-T lymphocyte immunoglobulin (ATG) is a potent inducer of iTreg. Lopez et al. [87] and Feng et al. [88] showed that thymocyte-induced ATG (thymoglobulin; Genzyme) converts human effector T cells into iTreg that subsequently suppress the proliferation of autologous responder cells to external stimuli. Ruzek et al. [89] further demonstrated that anti-mouse thymocyte globulin induces Treg in mice, which express several Treg markers (but not Foxp3) and inhibit GVHD. Similarly, we have recently shown that Fresenius anti-T lymphocyte globulin (ATG-F) can generate Treg *in vivo* that suppress mixed lymphocyte culture in patients undergoing allogeneic SCT [90]. These results may pave the way for novel therapeutic potentials; in addition, ATG may be synergized with another Treg inducer to obtain stronger long-term tolerance against the aggressive activity of T cells in allotransplantation and GVHD.

The majority of adoptively transferred Treg maintain their suppressive activity, but a minority of cells lose Foxp3 expression and can differentiate into Tcon [91]. It is conceivable that understanding why cells lose their “Treg-ness” and preventing this dedifferentiation *in vivo* will improve both the safety and efficacy of Treg therapy. Since Foxp3 is the master regulator of Treg function, alterations in Foxp3 expression or activity are likely involved in converting Treg to Tcon. Foxp3 expression is modulated by DNA methylation; therefore, administration of selective demethylating agents may enhance Treg function and fidelity *in vivo *[92].

In preclinical studies, Azacitidine (AzaC) treatment of allotransplanted mice mitigates deleterious GVHD while preserving beneficial GVT effect, by peripheral conversion of alloreactive effector T cells into Foxp3+ Treg and epigenetic modulation of genes downstream of Foxp3 required for the suppressor function of Treg [93]. Thus, the administration of AzaC after transplantation in humans may provide a simple and relatively nontoxic approach to limit GVHD while preserving the GVT effect and engraftment potential of donor T cells.

It is likely that different immunosuppressive and immunomodulatory agents will be more or less permissive for Treg development and function. For example, cyclosporine A (CsA) was shown to inhibit Treg function. Reduced suppressor function of CsA-exposed Treg was IL-2 dependent and correlated with a decreased number of Foxp3+ T cells both *in vitro* and *in vivo* [81]. On the other hand, these inhibitory effects by CsA on Treg function may have important implications for boosting immune responses to vaccination protocols.

Interestingly, the role of lenalidomide in the modulation of Treg remains unresolved. As discussed above, lenalidomide was shown to inhibit the proliferation and function of Treg *in vitro* [59]. Idler et al. [94] studied the changes in Treg population in patients with CLL treated with lenalidomide over a prolonged period of time and showed that lenalidomide decreased the percentage as well as the absolute number of Treg. However, a recent report by Lee et al. [58], as discussed above, showed that lenalidomide has a biphasic effect on Treg in CLL. Lenalidomide is highly effective in treating newly diagnosed and relapsed/refractory MM. Clinical data indicate that lenalidomide in combination with dexamethasone is highly effective in relapsed/refractory MM following allogeneic SCT, which is associated with an increase in Treg number [95]. However, lenalidomide maintenance after allogeneic SCT as part of first-line treatment in MM was not found feasible by the same group, mainly because of the rapid induction of acute GVHD [96]. Notably, Treg were increased at the ninth cycle, without correlation with clinical parameters. It was suggested that the Treg elevation occurred as a reaction against the immune-stimulating effects of lenalidomide. 

Raja et al. [97] have shown that the combination of lenalidomide and dexamethasone increases Treg in patients with previously untreated MM. The data suggest that, in spite of a positive antitumor immune response in patients treated with lenalidomide and dexamethasone, Treg are increased.

The conflicting but compelling data from *in vitro* studies that lenalidomide inhibits Treg suggest that the *in vivo* effects of lenalidomide might be a result of the microenvironment on the immune cells. Thus, the data may be interpreted such that, once lenalidomide induces effector immune responses *in vivo*, there is a negative feedback induced by transformation of the T helper cells into Treg to maintain the immune homeostasis [98].

## 9. Conclusions

Building on extensive research in Treg biology and preclinical testing of Treg therapeutic efficacy over the past decade, we are now at the point of evaluating the safety and efficacy of Treg therapy in humans. SCT seems a clinical setting suited to prove the efficacy of adoptive Treg therapies and may pave the way for further applications.

As discussed in our review, initial studies in clinical allogeneic SCT are very encouraging and have demonstrated that Treg-based clinical studies are feasible and do not result in toxicity. Human Treg infusion appears to suppress GVHD risk of Tcon while promoting enhanced immune reconstitution and decreasing the incidence of infectious complications. 

In patients with malignant diseases, a direct correlation between higher Treg numbers and more aggressive clinical-biological features, as well as with disease progression, has been described in several types of tumors. Elimination or inhibition of Treg might be particularly useful in the context of therapeutic vaccination against tumor-associated antigens, as stressed above. 

 Finally, further improvements in Treg *ex vivo* or *in vivo* expansion technologies and the use of costimulatory novel compounds to get higher cell yields, as detailed above, will “unlock the power” of Treg and facilitate the broader exploration of Treg therapies, for example, for the treatment of active GVHD or the prevention of graft rejection after solid organ transplantation.
